# Inflammatory Signaling in Hypertension: Regulation of Adrenal Catecholamine Biosynthesis

**DOI:** 10.3389/fendo.2018.00343

**Published:** 2018-06-28

**Authors:** Collin J. Byrne, Sandhya Khurana, Aseem Kumar, T. C. Tai

**Affiliations:** ^1^Department of Biology, Laurentian University, Sudbury, ON, Canada; ^2^Medical Sciences Division, Northern Ontario School of Medicine, Sudbury, ON, Canada; ^3^Department of Chemistry and Biochemistry, Laurentian University, Sudbury, ON, Canada; ^4^Biomolecular Sciences Program, Laurentian University, Sudbury, ON, Canada

**Keywords:** hypertension, adrenal medulla, catecholamine, immune, cytokine, glucocorticoid, epinephrine

## Abstract

The immune system is increasingly recognized for its role in the genesis and progression of hypertension. The adrenal gland is a major site that coordinates the stress response via the hypothalamic-pituitary-adrenal axis and the sympathetic-adrenal system. Catecholamines released from the adrenal medulla function in the neuro-hormonal regulation of blood pressure and have a well-established link to hypertension. The immune system has an active role in the progression of hypertension and cytokines are powerful modulators of adrenal cell function. Adrenal medullary cells integrate neural, hormonal, and immune signals. Changes in adrenal cytokines during the progression of hypertension may promote blood pressure elevation by influencing catecholamine biosynthesis. This review highlights the potential interactions of cytokine signaling networks with those of catecholamine biosynthesis within the adrenal, and discusses the role of cytokines in the coordination of blood pressure regulation and the stress response.

## Introduction

### Hypertension—the roles of catecholamines and inflammation

#### Hypertension

Approximately one in five Canadian adults live with hypertension; globally about 40% of adults over the age of 25 are hypertensive (1, 2). Recent guidelines have defined stage 1 hypertension as mean systolic blood pressure (SBP) ≥ 130 mm Hg or mean diastolic blood pressure (DBP) ≥ 80 mm Hg, and stage 2 hypertension as SBP ≥ 140 or DBP ≥ 90 mm Hg (3). Elevated blood pressure (BP) can cause changes in arterial structure that can increase risk of stroke, heart disease, kidney failure, and other diseases (4). Hypertension is a leading cause of renal failure, second only to diabetes (5). Globally, high BP is attributed to 54.5% of deaths caused by ischemic heart disease, 58.3% of deaths caused by hemorrhagic stroke and 50% of deaths caused by ischemic stroke (6). The significant health risks associated with hypertension have made it the world's leading risk factor for death, estimated as the cause of 13.5% of premature deaths (7). A majority of adults in both developing and developed countries have BP that is higher than is optimal and the risk of death from high BP is particularly great in lower income countries (4, 8). Globally, the cost attributed to BP above optimal levels, including both prehypertension and hypertension, is estimated at US$370 billion, roughly 10% of money spent on healthcare (9). Most recent estimates affirm that the treatment of hypertension is one of the most cost-effective approaches available for increasing quality-adjusted life-years and decreasing preventable deaths (10).

Hypertension can be classified into the categories of essential (or primary) and non-essential (or secondary) hypertension. Hypertension is diagnosed as essential when there is no discernable underlying cause. Essential hypertension is often attributed to a combination of genetic and environmental factors. Non-essential hypertension is directly linked to a pre-existing medical condition such as sleep apnoea, kidney damage, or diseases that consist of abnormal hormone biosynthesis (11, 12). Only a small minority (5–10%) of hypertension diagnoses are classified as non-essential, leaving the remaining majority (90–95%) of diagnosis to be classified as essential hypertension (13).

Like asthma, obesity, diabetes, and a multitude of other pathophysiological conditions, essential hypertension is a multigenic disease that is highly influenced by environmental factors (14). Multigenic traits involve multiple genes and do not have a single recognizable pattern of inheritance, as do single-locus Mendelian traits. Only a small proportion of cases of hypertension are directly caused by individual alleles, which show distinct inheritance patterns within families.

Recent estimates of hypertension awareness and control have demonstrated increased proportions of people who are aware of their condition, who receive treatment, and who have controlled their BP with medication (8). However, despite increased awareness and an abundance of available interventions, hypertension remains prevalent worldwide (6).

#### Regulation of blood pressure

**Figure 1:**
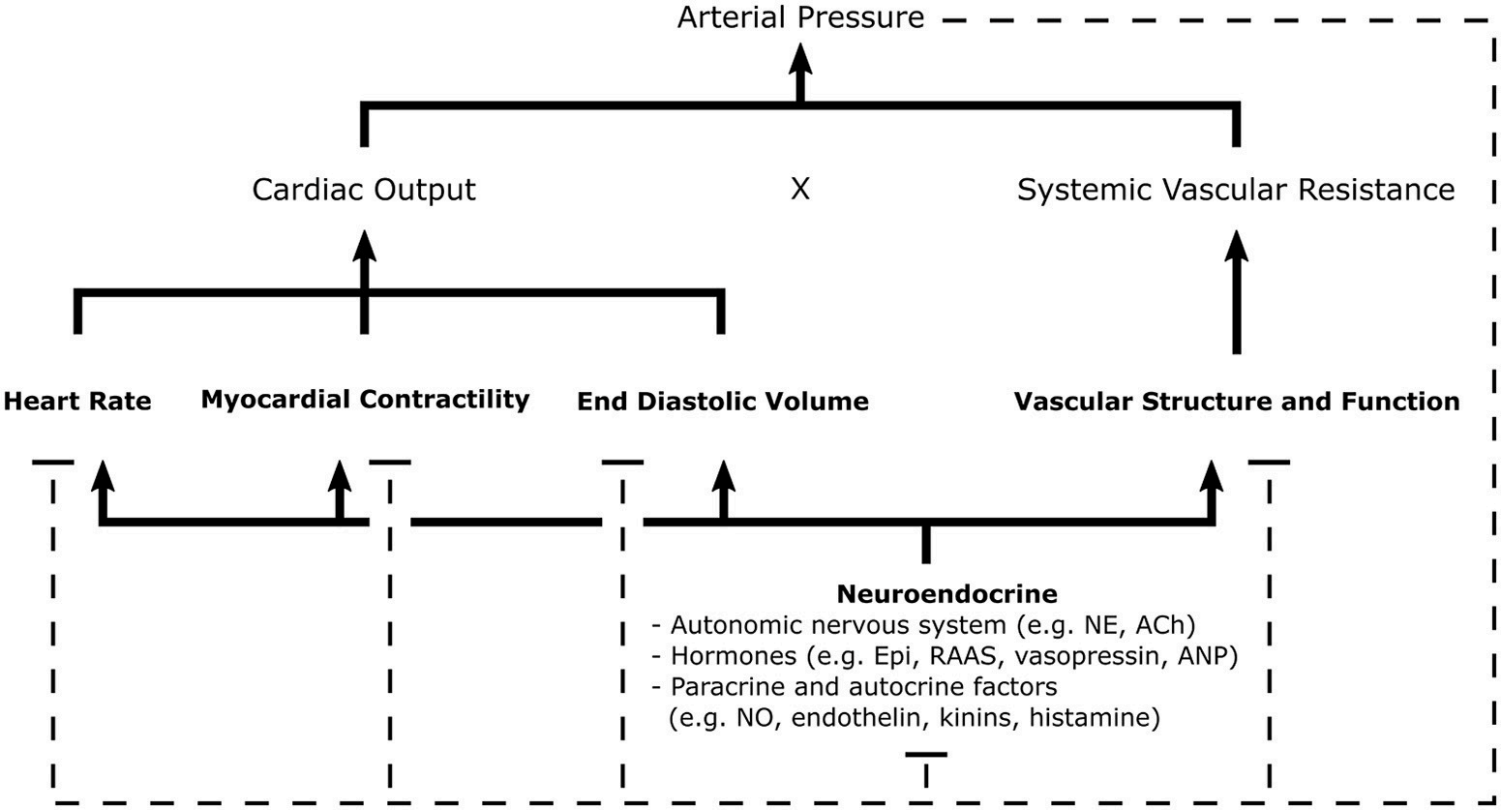
Schematic of the general mechanisms for blood pressure regulation. Arterial pressure is the product of cardiac output and systemic vascular resistance, parameters regulated by neuroendocrine signals which control cardiac, renal, and vascular function. Negative feedback pathways, depicted by dashed lines, are central to the maintenance homeostasis. Various sensors of arterial pressure mediate feedback by modulating sympathetic and parasympathetic tone; thereby, influencing many elements of cardiovascular function. The kidneys play a major role in the regulation of blood pressure through the RAAS, controlling pressure-natriuresis and pressure diuresis-mechanisms which determine fluid volume. Autocrine and paracrine mechanisms allow individual tissues to autoregulate vascular tone and blood flow through local release of vasoactive substances. Ach, Acetylcholine; ANP, Atrial Natriuretic Peptide; Epi, Epinephrine; NE, Norepinephrine; NO, Nitric Oxide; RAAS, Renin-Angiotensin-Aldosterone System [Concept derived from Cowley (15)].

There is a panoply of treatments available for reducing BP and combating hypertension. This is, in part, due to the numerous physiological parameters that influence BP and that are accessible targets for treatment. Blood pressure is the product of cardiac output and total systemic vascular resistance. These variables are dependent on parameters such as blood volume, extracellular fluid volume, arterial and venous compliance, and resistance to venous return (see Figure 1). Changes in the structure and function of kidneys, blood vessels, and the heart are regulated by neuroendocrine feedback mechanisms and serve to control blood volume and vascular resistance (16). Antihypertensive therapies must necessarily act upon the physiological mechanisms that control cardiac output and vascular resistance to effectively regulate BP.

The drugs available for treating hypertension fall into three general categories: diuretics (including thiazides, loop diuretics, aldosterone blockers, and potassium sparers), adrenergic inhibitors (including peripheral inhibitors, β-blockers, central α_2_-agonists, α_1_-blockers, and combined α- β- blockers), and vasodilators (including direct vasodilators, angiotensin-converting enzyme [ACE] inhibitors, calcium channel blockers, and angiotensin [Ang] II receptor blockers) (10). Further, recent efforts have been made to develop surgical interventions for treating hypertension when other approaches prove inadequate. These include renal sympathetic denervation and the implantation of devices which electrically stimulate activation of the carotid baroreflex (17, 18). Changes in lifestyle, when possible, are also effective measures for treating hypertension and are recommended in current treatment guidelines by expert committees (19–22). Some of these lifestyle changes include the consumption of a healthy diet, engaging in regular physical activity, minimization of alcohol consumption, maintenance of healthy body weight (BMI 18.5–24.9 kg/m^2^), maintenance of a moderate waist circumference (<102 cm for men, <88 cm for women), moderate sodium intake (<2,000 mg/day), and living in a smoke-free environment. Treatment recommendations for hypertension generally include a combination of antihypertensive drugs and appropriate lifestyle modifications, together with careful self-monitoring (3, 21).

#### Hypertension and catecholamine biosynthesis

Adrenergic inhibitors are useful pharmacologic agents for treating hypertension. Adrenergic signaling via α- and β-adrenergic receptors can influence cardiac output and peripheral resistance. The catecholamines (CAs) dopamine (DA), norepinephrine (NE), and epinephrine (Epi) are involved in the regulation of the cardiovascular system. These CAs are produced by neuroendocrine cells and have dual hormone and neurotransmitter functions.

The biosynthesis of CAs begins with the hydroxylation of tyrosine by the enzyme tyrosine hydroxylase (TH), producing L-3,4-dihydroxyphenylalanine (L-DOPA) (23). Next, L-DOPA is decarboxylated by the enzyme L-aromatic amino acid decarboxylase (AADC), converting it to DA (24). DA is then hydroxylated to produce NE; a reaction catalyzed by dopamine β-hydroxylase (DBH) (25). This sequence of reactions, which convert tyrosine to NE, is common in postganglionic neurons of the sympathetic nervous system and in specific brain regions such as the locus coeruleus. In adrenal chromaffin cells, and a small number of brain neurons, one additional biosynthetic step occurs, consisting of the methylation of NE by phenylethanolamine N-methyltransferase (PNMT) to produce Epi (26). Epi is the major secretory product of the adrenal gland, which is also the most abundant source of secreted Epi in the body (27, 28). CAs in the adrenal medulla are sequestered in CA storage vesicles of chromaffin cells. When stimulated, chromaffin cells release CAs from their vesicles through Ca^+2^-mediated exocytosis (29, 30).

Once released into circulation, CAs can interact with numerous adrenergic receptor types expressed in a variety of tissues. All CA receptors are G protein-coupled receptors (31). There are multiple forms of DA receptor, and they can be categorized in at least five (D_1−5_) different subtypes. Adrenergic receptor subtypes include α_1_-, α_2_-, β_1_-, β_2_-, and β_3_- adrenergic receptors, some of which can be divided into further subtypes. Adrenergic receptors are activated by the CAs Epi and NE, with each receptor having a distinct affinity for each ligand. Through these receptors, CAs can signal to numerous tissues throughout the body to produce a wide and coordinated physiological response.

The distribution and function of DA receptors suggests that DA may decrease BP by synergistically enhancing vasodilation, inhibiting synaptic NE release, decreasing circulating CAs, inhibiting aldosterone secretion and inhibiting sodium reabsorption in the kidney (32, 33). The α-adrenoceptors are important for the maintenance of vascular tone and promotion of smooth muscle contraction in other parts of the body. Sympathetic stimulation of α_1_-adrenoceptors is a major mechanism for sympathetic-mediated vasoconstriction (34). β-adrenergic receptors are expressed in airway smooth muscle, epithelium, endothelium, immunocytes, and myocardium (35). In cardiac tissue, although all three types are present, β_1_-adrenergic receptors are the major β-adrenoceptor type expressed. β_1_- and β_2_-adrenoceptor-mediated actions in the heart include positive inotropic (increased contractility), chronotropic (increased heart rate), dromotropic (increased conductivity), and bathmotropic (increased threshold of excitation) effects (36). β_3_-adrenoceptors require higher concentrations of CAs for activation than other β-adrenoceptors, and β_3_-adenoceptor signaling is suggested to counteract effects of β_1_- and β_2_-adrenoceptor activation, thus mediating a protective feedback loop to prevent adrenergic overstimulation.

Elevated plasma levels of Epi and NE have been reported in animal models of hypertension as well as in patients with essential hypertension (37–41). CA biosynthesis is dependent upon and correlated with the activity of the CA biosynthetic enzymes (40). Control of CA biosynthesis occurs primarily through the enzymes TH, DBH, and PNMT. AADC activity is typically high and is not rate limiting under normal physiological conditions (42, 43). In adrenal chromaffin cells, the expression of AADC is regulated to a lesser degree than the other CA biosynthetic enzymes (44–46). Regulation of TH, DBH, and PNMT is achieved through both transcriptional and post-transcriptional mechanisms (47–50). Transcript levels of TH and PNMT, and activities of TH and DBH are enhanced in the adrenal medullas of genetically hypertensive rat models (51–54). Further, PNMT is one of the putative gene loci linked to hypertension in genetic studies (55–57). Prolonged elevation of plasma CA levels can contribute to cardiac dysfunction by the over activation of vascular smooth muscle cells, resulting in ischemia and functional hypoxia; and oxidative damage (through the formation of oxidized CAs and oxygen free radicals), resulting in ultrastructural changes and cellular damage in cardiomyocytes (36). Oxidative damage may also lead to immune activation that contributes to the further progression of cardiovascular dysfunction (*vide infra*). Taken together, CAs play a role in hypertension, and the molecular mechanisms that regulate the CA biosynthetic enzymes and their activities are of interest for understanding the progression of hypertension and cardiovascular disease.

#### Hypertension and inflammation

The role of inflammation in the genesis of hypertension and accompanying organ damage is well established (16). Inflammation is one of the most important factors contributing to cardiovascular risk; and it is a major contributor to the formation, progression and destabilization of atherosclerotic lesions (58–60). The link between immune and cardiovascular function is apparent in major immune diseases including rheumatic diseases, HIV, and psoriasis. Cardiovascular pathologies are the leading cause of premature mortality in patients with autoimmune rheumatic diseases (61). Individuals with HIV infection have higher risk of cardiovascular disease (CVD), arterial stiffness, systolic, and pulse pressures than matching uninfected individuals (62). A recent meta-analysis of observational studies concluded that psoriasis, a chronic inflammatory skin condition, is associated with increased prevalence and incidence of hypertension and that odds of hypertension increase with severity of psoriasis (63). Inflammation is an essential component of many diseases, and the connections between innate and adaptive immunity, hypertension, and CVD add support to the role of the immune system in cardiovascular pathology (64).

A prospective cohort study of 20,525 women concluded that high plasma levels of the inflammatory biomarker C-reactive protein are predictive for the development of hypertension (65). Several studies have supported immune involvement in the elevated BP of spontaneously hypertensive rats (SHR). Purcell and Gattone (66) found that young SHRs have an elevated rate of nerve growth into the thymus, a primary lymphoid organ important in T-cell development, compared to their normotensive Wistar Kyoto (WKY) counterparts. Others have found that treatment of SHR with antithymocyte serum or with the immunosuppressant cyclophosphamide lowers BP (67, 68). Later studies established the role of the adaptive immune response in hypertension after finding that mice with a genetic deletion in recombinase-activating protein (RAG-1^−/−^), which lack T- and B-lymphocytes, experience blunted hypertension in response to both Ang II and deoxycorticosterone acetate (DOCA)-salt; adoptive transfer of T-cells restored the elevation in BP (69). This study also identified the role of the cytokine TNF-α in BP elevation when mice treated with Ang II responded with both increased BP and increased synthesis of TNF-α from T-cells; anti-TNF-α therapy with etanercept (a TNF-α inhibitor) blunted Ang II-mediated elevations in BP (69). Taken together, these studies suggest the potential for enhanced neural activation of T-cells in hypertension as well as a functional importance of cytokine signaling in BP regulation. Also supporting the role of cytokines in hypertension, multiple studies have identified altered profiles of pro- and anti-inflammatory cytokines and cytokine production capacity in humans when comparing hypertensive or pre-hypertensive patients to control subjects (70–73). Although results are sometimes conflicting, these studies implicate cytokines such as IL-1, IL-4, IL-6, IL-7, IL-13, TNF-α, and CCL2 in human hypertension. Other cytokines not analyzed or not changed in circulating concentration may also be important in hypertension because of the potential for local regulatory effects at important centers for BP regulation.

Introduction of exogenous cytokine has been reported to induce changes in BP. The cytokines IL-2 and IL-10 predominantly decrease BP whereas the cytokine IL-6 predominantly increases BP (74–79). Other cytokines, such as TNF-α and IL-1, appear to have more intricate effects in relation to BP regulation. Regulation of BP by cytokines may be mediated by signaling to neural control centers or by direct actions on peripheral tissues. In a screening experiment for changes in chemokine expression in DOCA/salt-induced hypertensive mice, transcript of the chemokine CCL2 and its receptor CCR2 were increased in aortas after the onset of hypertension. Treatment of mice with the CCR2 antagonist, INCB3344, substantially reduced DOCA/salt-induced infiltration of macrophages in aortic wall and DOCA/salt-induced elevations in BP (80). IL-1 can modulate BP by influencing activity of neurohormonal control centers in the brain (81–84). IL-1 can also modify vascular reactivity to NE (85). Human cancer patients who receive high doses of IL-2 demonstrate hemodynamic changes which suggest decreased peripheral resistance but increased cardiac output, with an overall reduction in mean arterial pressure (86, 87). Similar hemodynamic changes and hypotension are observed in experimental animals (88, 89). Interestingly, IL-2 can decrease circulating Epi in mice, and reversal of IL-2-induced hypotension is associated with a rise in circulating CAs (89). Like IL-2, experimentally-induced elevations in IL-10 levels are associated with decreases in BP in animal models (82, 90, 91). IL-2 and other cytokines may reduce CA-mediated vasoconstriction by enhancing superoxide generation, a molecule that can interact with CAs and inhibit their signaling function (92–94). In contrast to many other cytokines, IL-6 appears to increase BP. In a large cohort study of healthy men, after controlling for age and other cardiac risk factors, baseline plasma IL-6 levels were positively associated with increased systolic and diastolic BP, pulse pressure, and mean arterial pressure (95). In animal experiments, IL-6 is shown to contribute to Ang II-induced, but not salt-sensitive hypertension (75, 76, 96, 97). Pro-hypertensive effects of IL-6 may be mediated, in part, by vasoconstrictive effects of the cytokine that could result in increased peripheral resistance (85, 98). The major proinflammatory cytokine TNF-α can induce hypotension; however, inhibition of TNF with etanercept can prevent elevations in BP caused by Ang II infusion, high-fructose feeding, and chronic inflammation in animal models (69, 99–102). TNF-α inhibition does not influence BP in salt-dependent hypertension (103). TNF-α can directly modulate arterial tone, and TNF-mediated signaling is involved in cardiac remodeling (85, 98). Prenatal exposure to elevated levels of cytokines may also contribute to hypertension during adulthood (104). This may involve morphological changes in tissues, as suggested by findings of increased adrenergic innervation of the thymus in humans provided with IFN-α therapy (105). The BP-regulatory effects of cytokines are complex, affecting multiple physiological systems in combination with hormones, neurotransmitters, and other signaling molecules. Future investigations into the integration of cytokine signaling with the other BP regulatory systems will help to illuminate the events responsible for BP dysregulation in disease.

**Figure 2:**
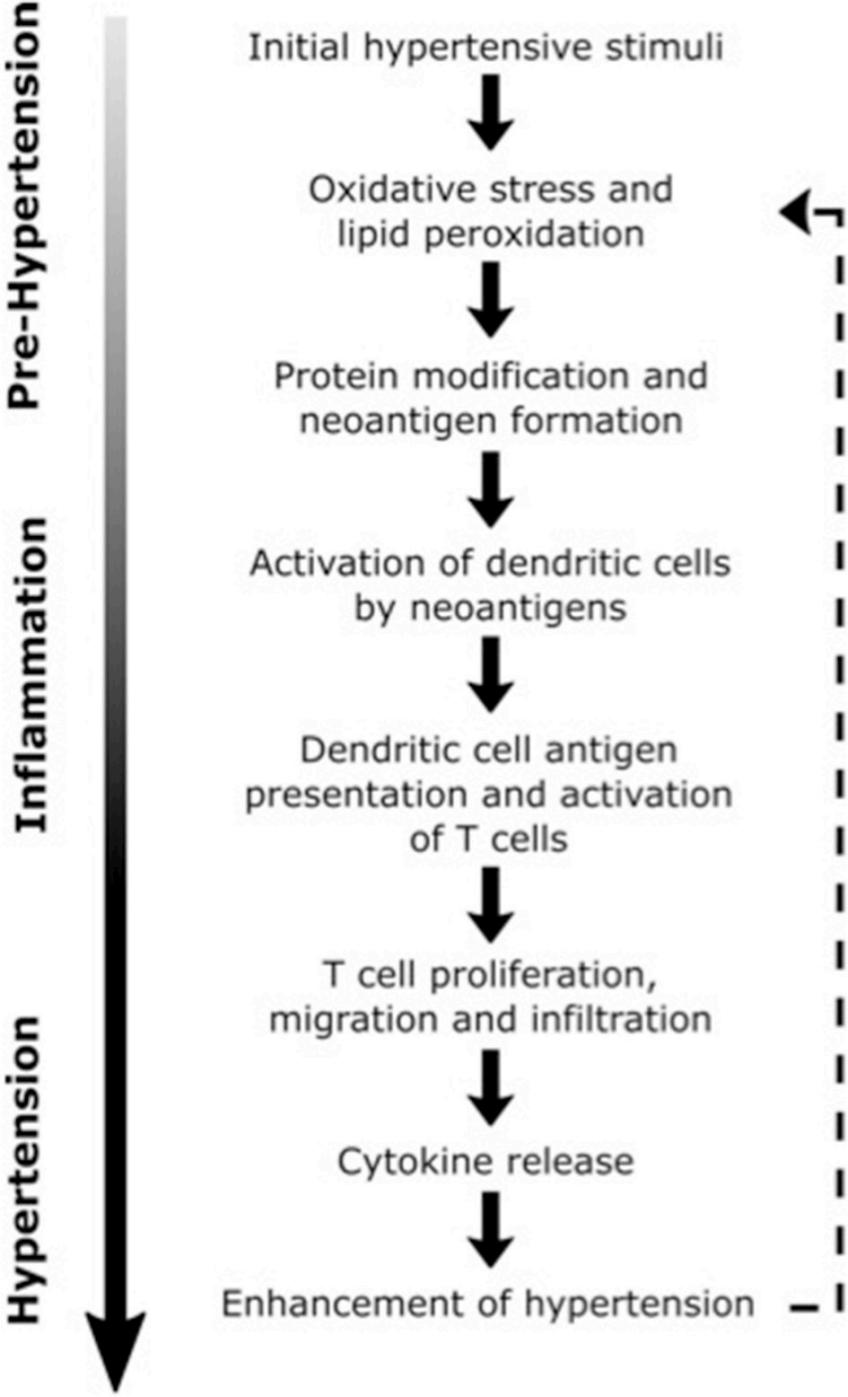
Possible inflammatory processes contributing to the progression of hypertension. Dashed line illustrates the positive feedback loop that may lead to further elevations in blood pressure [Concept derived from Harrison (106)].

In describing a model for the interaction of inflammation and hypertension, Harrison et al. (106) hypothesized that modest elevations in BP (to values of SBP ~135–140 mm Hg), largely caused by activity of the CNS, trigger immune changes that lead to hypertension (see Figure 2) (106). In this model, initial elevations in BP are responsible for neoantigen formation from oxidation and altered mechanical forces in vasculature. Neoantigens then induce inflammatory responses in the kidneys, blood vessels and other tissues, where lymphocyte infiltration and expression of inflammatory mediators such as CCL2, IL-1, IL-6, IL-17, and TNF-α is increased (69, 107–109). These cytokines and inflammatory mediators work in concert with CAs and other BP-elevating hormones leading to vascular and renal dysfunction and initiating a more severe hypertensive state (106). This feed-forward model described by Harrison et al. (106) was foretelling of findings by Kirabo et al. (110), whose work outlined a mechanism for hypertension based on an autoimmune-like reaction. In this mechanism, initial increases in BP lead to oxidative stress and lipid peroxidation which results in neoantigen formation, initiation of T-cell proliferation and cytokine biosynthesis, and further increases in BP. Recent studies also demonstrate that immune cell function can be altered by high salt microenvironments in the skin and skeletal muscle, with salt acting as a proinflammatory stimulus for the development of hypertension (111, 112). With the support of these and other findings, a new paradigm is being established that implicates inflammation in the elevation of BP and progression of hypertension.

### The major mechanisms of adrenal medullary regulation

The adrenal gland is a key organ involved in the physiological adaptation to stress. The “fight-or-flight” response, first described by Cannon in the early twentieth century, is characterized by increased BP, increased heart rate, increased cardiac output, and changes in vascular and respiratory smooth muscle tone (113, 114). The two major hormones secreted into circulation that facilitate the physiological stress response include cortisol and Epi, both being primarily products of the adrenal cortex and medulla, respectively (28). There are two major effector circuits that are activated when the CNS perceives or anticipates a stress. They include the hypothalamic-pituitary-adrenal (HPA) axis, which stimulates the adrenal medulla through a hormonal mechanism, and the sympathetic-adrenal (SA) axis, which stimulates the adrenal medulla through a neural mechanism (115). These axes are in many ways physically distinct but they also have overlapping CNS components and physiological functions. Initiation of the physiological stress response, involving either HPA or SA axis, is primarily derived from structures of the limbic system. Termination of the stress response, caused by hormonal and neural feedback, also involves many of these same limbic structures. Integration of hormonal and neural signaling cascades allows the HPA and SA axis to function cooperatively while also tailoring individual responses to the specific initiating stimuli (116).

#### Hypothalamic-pituitary-adrenal axis

**Figure 3:**
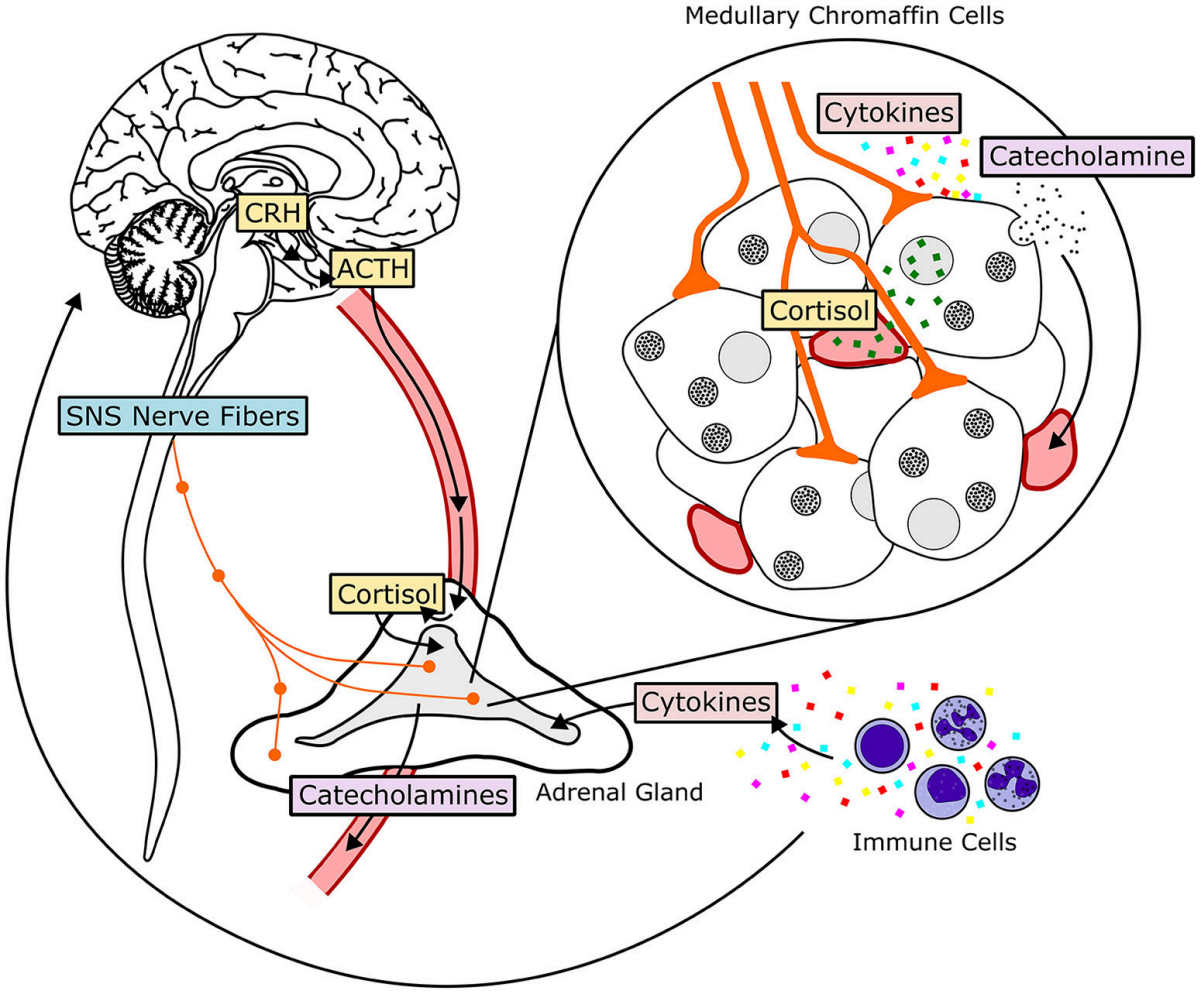
Hormonal and neural mechanisms regulating adrenal medullary chromaffin cells. The HPA-axis, comprised of the hormones corticotropin-releasing hormone (CRH), adrenocorticotropic hormone (ACTH), and cortisol, is shown in yellow. The SA-axis, comprised of afferent preganglionic sympathetic nervous system (SNS) fibers, is shown in blue. Green squares represent glucocorticoid (cortisol) produced in the adrenal cortex and traveling to the adrenal medulla through vasculature. Acetylcholine, pituitary adenylate cyclase-activating peptide, and other neurotransmitters are released from synaptic terminals. Cytokines, transported to the adrenal medulla or produced locally, influence adrenal chromaffin cell function and response to HPA- and SA-axis activation. Both glucocorticoids and sympathetic input stimulate release of catecholamines, primarily epinephrine, from chromaffin cells by exocytosis. Epinephrine then enters systemic circulation and travels to target tissues throughout the body.

The HPA axis consists of the paraventricular nucleus (PVN) of the hypothalamus, the anterior pituitary gland and the adrenal gland (117). The HPA axis is activated when afferent neurons from multiple brain regions stimulate hypophysiotrophic neurons of the paraventricular nucleus, inducing them to release corticotropin-releasing hormone (CRH), and vasopressin (see Figure 3). CRH and vasopressin then travel through hypophysial portal vessels to the anterior pituitary. The axons of CRH neurons present in the PVN project to the median eminence through the lateral retrochiasmatic area. CRH released from the outer layer of the median eminence binds to receptors on pituitary corticotropes and promotes the secretion of adrenocorticotrophic hormone (ACTH) into systemic circulation (118, 119). In the presence of CRH, vasopressin has a synergistic effect, enhancing secretion of ACTH into circulation. ACTH then travels to parenchymal cells of the adrenocortical zona fasciculata, where it binds to plasma membrane receptors and initiates a rapid increase in the biosynthesis and secretion of glucocorticoids (GCs). Once in systemic circulation, GCs bind to ubiquitously expressed intracellular glucocorticoid receptors (GRs) to induce physiological adaptations to the initial stressor. An intra-adrenal portal vascular system allows the exposure of adrenal medullary cells to especially high concentrations of GCs released from the adrenal cortex (120). GCs produce their cellular effects primarily by regulating transcription. Endogenous cortisol (corticosterone in rodents) is a lipid-soluble steroid hormone that binds to the cytoplasmic GR. Prior to ligand binding, GR is located in the cytoplasm as a multiprotein complex (121). HSP90, one of the proteins in this complex, maintains the cytoplasmic retention of GR until binding of a ligand to GR causes dissociation from the complex and translocation into the nucleus. GR then homodimerizes and binds to glucocorticoid response elements (GRE) in the promoter regions of target genes directly, or interacts with other transcription factor proteins causing transactivation or transrepression (121). An alternate manner in which GCs can initiate transcription or signaling events is via membrane receptor activation, which, when coupled with G-proteins can induce downstream signaling cascades. This alternate mechanism of signaling activation triggered by GCs is exerted in situations wherein rapid functional changes associated with GC mediated signaling activation are critical (122).

GCs are important regulators of homeostasis during basal conditions and during stress. They are critical for regulating cardiovascular, immune, metabolic, developmental, and reproductive processes (117). For example, the strong influence of GCs on immune function has allowed them to become some of the most commonly used compounds for therapeutic treatment of inflammatory, autoimmune, and lymphoproliferative disorders. In human peripheral blood mononuclear cells, ~20% of genes are regulated either positively or negatively by GCs (123). The effects of GCs on cardiovascular regulation are also important. Cortisol is a regulator of BP in humans and can lead to hypertension when in excess (124). Chronically elevated GC, either endogenous (as seen in cases of adrenal hyperplasia or dysplasia or elevated ACTH expression), or by exogenous means (when administered for immunosuppressant therapy), can cause a clinical disorder known as Cushing syndrome. It is estimated that 80% of patients with endogenous elevated GCs, and 20% of corticosteroid therapy patients with Cushing syndrome present with elevated BP (125, 126).

One way that GCs can influence BP is by influencing CA biosynthesis and secretion. GCs directly increase the release of CAs by sympathetic nerves and adrenal medullary cells (127, 128). Early evidence of GC control over adrenal CA biosynthesis was demonstrated in experiments performed by Wurtman and Axelrod (129), who reported that ablation of the pituitary gland decreases PNMT activity, which can be restored by addition of ACTH or GC (129). Later, *in vitro* and *in vivo* experiments confirmed that GCs are responsible for increasing PNMT mRNA expression (128, 130, 131), increasing the amount of functional intronless mRNA splice variant (49), increasing PNMT activity (49), and enhancing PNMT protein stability via regulation of the co-substrate S-adenosyl-methionine (132–134) in adrenal chromaffin cells. Although, Greene and Tischler (135), previously thought that PC12 cells do not synthesize PNMT or epinephrine, and are primarily noradrenergic, later studies by Kim et al. (136) and Byrd et al. (137) showed that these cells do indeed express low levels of PNMT and Epi, and the expression of both is significantly increased in the presence of the synthetic GC, dexamethasone (135–137).

Studies in rat pheochromocytoma cells show that, in addition to PNMT, GCs regulate the other CA biosynthetic enzymes to produce parallel increases in their transcript level and activity (136, 138–141). Similar observations pertaining to regulation of enzyme transcript levels have also been made with primary cultures of bovine adrenal medullary cells; however, unlike rat pheochromocytoma cells, in bovine chromaffin cell primary cultures DBH transcript does not appear to be regulated by GC (50, 142).

Thus, GCs can increase transcript of TH, DBH, and PNMT. The site critical for GC responsiveness of the rat TH gene is located at about −5.7 kb and it closely resembles the activator protein 1 (AP-1) binding site (143). This finding is consistent with earlier observations that the proximal promoter region (−773 to +27 bp) is not sufficient for GC regulation of the TH gene (138, 139). Another functional GRE has been identified at ~2.4 kb upstream in the mouse TH promoter (144). Several putative GREs have been identified in the first 1 kb of the upstream rat DBH gene, with corresponding sequences in the human DBH promoter (140). Although long exposure with GCs can increase transcript levels of DBH in PC12 cells, functionality of putative GREs in the DBH promoter has not yet been proven (140). GCs are also important regulators of PNMT transcription (131). Three functional GREs have been identified in the proximal 1 kb rat PNMT promoter, and activation at these sites can be synergistically regulated by the transcription factors early growth response 1 (Egr1) and activator protein 2 (AP-2) (145–147).

#### Sympathetic-adrenal axis

Working alongside the HPA-axis, the SA-axis, consisting of the direct innervation of adrenal medullary chromaffin cells by the sympathetic nervous system, also signals the adrenal medulla to synthesize and secrete Epi (148). Stress signals, primarily originating from limbic structures, are transmitted to preganglionic sympathetic neurons in the intermediolateral cell column of the thoracolumbar spinal cord which project, via the splanchnic nerve, to chromaffin cells of the adrenal medulla (116). The cortex is also innervated by the splanchnic nerve and neurotransmitters such as acetylcholine (ACh), released at the adrenocortical junction, regulate steroid biosynthesis and can influence vasculature to regulate adrenal perfusion (149–152). The neural stimulus is delivered to each chromaffin cell by several synaptic boutons and compelling evidence now suggests that gap junctions also help to propagate electrochemical signals between neighboring adrenocortical cells (153, 154). A combination of neurotransmitters and neuropeptides such as neuropeptide Y (NPY), acetylcholine (ACh), and vasoactive intestinal peptide (VIP) are released from sympathetic nerve terminals and bind to plasma membrane receptors on chromaffin cells. These substances stimulate the release of large amounts of stored CAs from chromaffin cell vesicles via Ca^+2^-mediated alteration of action potential and exocytosis; the frequency of these action potentials is dependent on the concentration of ACh (155–160). ACh directed CA secretion can also be mediated in the presence of K^+^ and Na^+^ induced membrane depolarization (161, 162). Additionally, the adrenal cortex receives input from medullary ganglion cells that synthesize NE, NPY, and VIP, amongst other biomolecules; this paracrine interaction can also influence steroidogenesis (149). The extrinsic innervation of the adrenal gland, and intrinsic neural networks within it allow for an integrated signaling and fine tuning of adrenal function (150, 163). Due to the direct innervations of adrenal chromaffin cells, the SA effector circuit has a short latency in comparison to excitation via the HPA axis, which is generally longer lasting and slower to respond (164). Stimulation of chromaffin cell activity by the SA axis may contribute to hypertension through either an increase in sympathetic nerve firing or an unusually high sensitivity of chromaffin cells to sympathetic stimulation (165–168).

Synaptic transmission at the SA synapse is mediated by the small molecule transmitter acetylcholine (ACh) and by neuroactive peptides. The frequency of action potential firing at sympathetic nerve terminals influences the types of neurotransmitters released from the presynaptic nerve at the SA synapse. Stress is associated with high frequency splanchnic nerve firing, whereas basal sympathetic tone is characterized by lower frequency firing (169). In the preganglionic sympathetic nerves at the SA synapse, small synaptic vesicles (SSVs) contain ACh and large dense core vesicles (LDCVs) contain neuropeptides such as pituitary adenylate cyclase-activating peptide (PACAP). During high frequency firing both LDCVs and SSVs are released from the presynaptic nerve terminals. During basal conditions, only SSVs are released (170). Both PACAP and ACh are integral at the SA synapse for promoting CA biosynthesis and secretion (115).

ACh is perhaps the best characterized molecule for synaptic transmission from the splanchnic nerve to the adrenal medulla. ACh binds to both nicotinic and muscarinic plasma membrane receptors on chromaffin cells (mAChRs and nAChRs, respectively). nAChRs are also classified as muscle or neuronal nAChRs depending on their site of expression. Although both AChR types can promote CA release, the reliance on stimulation via mAChRs or nAChRs is species dependent. For example in bovine adrenals, nAChRs are primarily responsible for cholinergic transmission and CA release, in chickens, the mAChRs are the key players, whereas in other species it could be both (142, 171–173). Structurally, neuronal nAChRs are heterodimeric proteins made of 5 subunits, two α, and three β subunits, combinations of which can give rise to numerous receptor subtypes, with the α3β4 being the most pertinent nAChR for CA secretion (172). The nAChRs are ligand gated cation channels mediating quick excitation responses, while mAChRs are metabotropic receptors coupled with G-proteins resulting in slower neuronal signaling (172, 173). There are 5 isoforms of the mAChRs, M_1_-M_5_, which are species specific and are coupled with different Gα proteins and signaling pathways (173).

Activation of nAChRs increases TH mRNA in chromaffin cells in a protein kinase A (PKA)-dependent manner (142, 174, 175). Cholinergic stimulation of chromaffin cells also induces PNMT promoter-driven luciferase activity through a PKA-dependent mechanism (176). Additionally, *in vitro* and *in vivo* evidence supports the role of mAChRs in activating PNMT expression, via induction of the transcription factor Egr1 (177, 178).

Evolutionarily conserved, PACAP belongs to the vasoactive intestinal protein (VIP) family of peptides. PACAP is primarily released from LDCVs during high frequency neuronal firing, and is important for generating sustained increases in CA synthesis and secretion by chromaffin cells (179, 180). The PACAP precursor is processed into two bioactive forms, namely PACAP38 and PACAP27. The PAC1 receptor (PAC1R), which belongs to the subclass B1 GPCR, is selective for PACAP38/PACAP27, while VPAC1 and VPAC2 have affinities for both PACAP and VIP (181). PAC1R signals through Gαs, which regulates adenylyl cyclase (182). Binding of PACAP to PAC1R can signal through the conventional cyclic adenosine monophosphate (cAMP)-PKA pathway and at least two other insulated, cAMP-sensitive signaling pathways involving the signal transduction proteins exchange protein directly activated by cAMP (Epac) and the extracellular signal regulated kinases (ERK) 1 and 2 (183–185). The PAC1R can also stimulate Gαq, which activates a phospholipase C (PLC)-protein kinase C (PKC) pathway (186).

PACAP is now recognized as a critical peptide for signaling at the “splanchnicoadrenomedullary” junction under situations of stress (187, 188). PACAP is capable of upregulating chromaffin cell expression of TH, DBH, and PNMT transcripts (189–191). In studies using PACAP^−/−^ mice, the biosynthesis of TH and PNMT transcripts was significantly reduced in animals exposed to restraint stress, possibly due to blunted Egr1 and cFos; under sustained stress, reduction in CRH mRNA in the PVN and circulating corticosterone was observed indicating that PACAP is critical in the stress response (190, 192). In addition to regulation of CA biosynthesis, other studies have demonstrated the importance of PACAP in regulating CA secretion from adrenal cells, and its role in nerve firing (180, 184, 193, 194). Taken together, these studies suggest a central role for PACAP in HPA axis function during stress. Consistent with this role, disrupted PACAP signaling has been correlated with anxiety, depression, behavioral and cognitive changes, and other psychopathologies (195–198).

As mentioned above, signaling via cAMP is an important molecular mechanism induced by both ACh and PACAP, and is involved in the regulation of CA biosynthetic enzymes in adrenal chromaffin cells. In primary cultured bovine adrenomedullary chromaffin cells, cAMP signaling produces synchronized increases in both transcript and activity levels of TH, DBH, and PNMT (27). Similar activation of the CA biosynthetic enzymes by cAMP signaling occurs in rat chromaffin cells (138–140, 176, 199). It should be noted that in both rat and bovine models, the induction of PNMT by cAMP is relatively small compared to the induction of TH and DBH. Signaling by cAMP activates PKA and can lead to tissue-specific induction of other signaling pathways. For example, in PC12 cells, PACAP activates PKA signaling as well as signaling through the mitogen-activated protein kinases (MAPKs) p38 and ERK1/2 via a PKA dependent mechanism (200, 201). Signaling by ERK1/2, downstream of PKA activation, may contribute to PNMT transcriptional activation (191).

The promoters of TH, DBH, and PNMT all contain motifs that can bind many common transcription factors. The transcription factors specificity protein 1 (Sp1), AP-2, and Egr1 all have functional consensus sequences in the promoters of TH, DBH, and PNMT rat genes. AP-1 and cAMP-responsive element (CRE) motifs are also present within the rat TH and DBH promoters (155, 199). Induction of Egr1 in rat chromaffin cells occurs through a cAMP/PKA signaling mechanism (202, 203). In rat chromaffin cells, Egr1 regulates transcription of TH, DBH, and PNMT (199, 204, 205). Splanchnic nerve activation, the cholinergic agonists nicotine and muscarine, and the neuropeptide PACAP are all inducers of Egr1 (176, 177). Transcriptional activation of TH and DBH also occur through cAMP induction of transcription factor binding to AP-1 and CRE motifs (206–209). Other transcription factors potentially involved in the activation of CA biosynthetic enzymes by neural stimuli include Sp1 and AP-2 (176, 191). Taken together, these studies suggest that the neuronal regulation of chromaffin cells involves a number of transcription factors which can act individually, or cooperatively to regulate expression of the enzymes responsible for CA biosynthesis.

#### Chronic stress and hypertension

The coordinated functionality of both HPA and SA axes is essential for BP homeostasis. The functional balance between these axes is critical in the biosynthesis of CAs and in the maintenance of BP (116, 210). Exposure to chronic stress, however, leads to sustained elevated blood levels of CAs that can have adverse affects on health and have been correlated with hypertension (211–213). Studies of panic disorder, and chronic mental stress have documented co-morbidity of these conditions with hypertension, and reported elevated circulating cortisol and Epi, and an increased expression of PNMT in the sympathetic nerve fibers of affected patients (214, 215). Chronic stress results in SA overdrive thereby altering adrenergic output; this consequently elevates BP causing physiological and metabolic changes (216, 217). Repeated exposure to stress elevates Epi, and is correlated with hypertension (41, 218, 219). The extent of Epi activation is dependent on the type of stressor and duration of stress exposure (155, 220). Psychological stress has been correlated with elevated cortisol and Epi, with consequences on metabolism and physiological functions; this has been reported in people who experience chronic work stress, and consequently develop metabolic syndrome (221, 222). Indeed, stressors such as mood disorders, low social support, socioeconomic background etc. are factors that can influence cardiovascular health; effective strategies to manage cardiovascular health should, if possible, encompass interventions that target these psychosocial stressors (223, 224). Individuals with anxiety, especially as seen in those who experience post-traumatic stress disorder, are susceptible to developing hypertension, and have an elevated risk for other cardiovascular pathologies, possibly due to over activity of the HPA (225–227). Additionally, other factors such as physical fitness, diet, smoking, age, sex, and obesity can influence the onset and/or severity of hypertension (3, 228–230).

## Cytokine-mediated regulation of catecholamine biosynthesis

Investigations into the potential role of cytokines in regulating CA biosynthesis by the adrenal gland were, in part, inspired by insights gained from studying depression (231). Depression can be induced by alterations in NE and other neurotransmitter levels, and sympathetic hyperactivity is a well characterized attribute of the condition (232). It has also been reported that a large proportion of patients receiving IFN-α therapy for treatment of cancer or infectious disease develop a behavioral syndrome that is very similar to major depression (232). This finding led to questions about the influence of cytokines on neurotransmitter synthesis, and the role of cytokines in regulating neural activity. Interestingly, depression is now associated both with elevations in plasma levels of proinflammatory cytokines and increased risk of hypertension, cardiovascular morbidity, and mortality (233–235). Although the causal relationships are not yet resolved, possible influences of inflammatory mediators such as cytokines on catecholaminergic cell function are now being investigated for their contribution to hypertension and CVD.

In humans, treatment with IFN-α increases circulating levels of NE and Epi (236, 237). Both intravenous and intracerebroventricular administration of IL-1 to rats has been reported to increase plasma levels of NE and Epi, along with increased renal sympathetic nerve activity, SBP, and heart rate (238, 239). Central administration of IL-1 to rats has also been reported to increase ACTH secretion (240). These findings suggest that IL-1 can activate SA and HPA axes by direct stimulation of regulatory centers within the brain. In humans, peripheral administration of IL-6 increases plasma cortisol and NE but does not affect plasma Epi levels (241–244). Studies have suggested that peripherally, but not centrally administered, TNF-α elevates plasma CA levels in rats (245, 246). Increased expression of IL-10 in the brain can inhibit elevations in plasma NE resulting from myocardial infarction in rats (247). Numerous cytokines, including IFNs, IL-1, IL-2, IL-6, and TNF-α induce changes in brain CA synthesis or metabolism. Often, excitatory or inhibitory effects of cytokines in the brain are regionally dependent. Many of these same cytokines also modulate CA levels in the hypothalamus and influence function of the HPA axis (248, 249). For example, central and peripheral administrations of IFN-α both alter levels of DA and NE in specific regions of the brain (250–252). The patterns of altered CA levels differ depending on the location, central or peripheral, of IFN-α administration. This suggests that direct and indirect sensing of cytokines by the brain induce unique responses in CA synthesis by neural tissues. Numerous studies report similar regulatory effects for other cytokines in relation to brain CA synthesis. In peripheral tissues, the effects of centrally or peripherally administered cytokines on CA levels and CA turnover is tissue-specific, suggesting that cytokines can influence sympathetic activity both directly and indirectly, and that modulation of sympathetic nerve activity is specific rather than global (253–259).

Cytokines have also been reported to regulate CA biosynthetic enzymes *in vivo. In vivo* studies using rats demonstrate that the cytokines IFN-α, IL-1β, and TNF-α regulate the CA biosynthetic enzyme TH in catecholaminergic cells of the brain and carotid body (251, 260–262). Interestingly, centrally administered cytokines can regulate CA biosynthetic enzymes in the adrenal medulla as well, likely through indirect mechanisms involving neural activation at the level of the CNS and downstream effects mediated by the HPA or SA axes (263). For a more complete presentation of cytokine effects in the brain, HPA and SA axis, several reviews are available (248, 249, 264, 265). In sites of CA biosynthesis outside the brain, the influence of cytokine signaling is only beginning to be understood.

### Cytokine expression by adrenal chromaffin cells

Adrenal cytokines can originate either systemically or locally; both situations have possible importance to cytokine-mediated regulation of adrenal function during hypertension. Numerous studies have identified unique profiles of circulating and tissue-expressed cytokines in hypertensive animal and human subjects (73, 80, 82, 95). Even during normal physiological conditions, cytokines are expressed at detectable levels by adrenal medullary tissue. Cytokines are expressed at varying levels throughout the adrenal gland (266, 267). The highest levels of expression are most commonly observed in the cortex or steroid-producing cells within the medulla, although expression of cytokines by chromaffin cells themselves has also been demonstrated in a number of studies (see Table 1). In humans, as in many species, the adrenal medulla is contiguous with the adrenal cortex, meaning that chromaffin cells are in direct contact with steroidogenic cells (303). Chromaffin cells are also receptive to many cytokines that are produced locally in the adrenal gland (see Table 1). Receptiveness to cytokines is demonstrated either by expression of cytokine receptors or by response of isolated chromaffin cells to cytokines. In instances where the cytokine and its receptor are co-expressed, or when a locally produced cytokine can elicit a response in chromaffin cells, there is a possibility of autocrine or paracrine signaling that may influence endocrine function of the adrenal medulla (317).

**Table 1:** Cytokine expression, responsiveness, and signaling observed in adrenal gland.

**Cytokine**	**Cytokine Expression (organism, tissue)**	**Responsiveness (organism, tissue, mode of exposure)**	**Cytokine Receptors (receptor name, organism, tissue)**	**Supported Signaling Mechanisms**
IFN-α		Bovine (medulla and chromaffin cells, *in vitro*) (268, 269, 270)	IFNAR2; Bovine (chromaffin cells) (271)	PKC, ERK1/2, STAT 1 and 2 (268) PKC (270)
IL-1 α/β	Human (cortex, medulla, and KAT45 cells) (272, 273) Rat and Mouse (chromaffin cells) (274) Rat (cortex and medulla, chromaffin, and PC12 cells) (275, 276, 277, 278, 279)	Human (chromaffin and KAT45 cells, *in vitro*) (280, 272) Rat (medulla, *in vivo*) (275) Rat (adrenal and PC12 cells, *in vitro*) (281, 282, 283, 284, 285, 286) Mouse (primary chromaffin cells, *in vitro*) (287) Bovine (medulla and chromaffin cells, *in vitro*) (288, 289, 290, 291)	IL-1R1; Rat (medulla and PC12 cells) (275, 292) IL-1R2; Rat (medulla) (293) IL-1RA; Rat (chromaffin cells) (279)	MAPK, NO/PKC, NO/GC, NPY, PKA/NO (280) NPY (287) ERK1/2 (288) Ca^2+^ (292) PKA (282) CRH (272)
IFN-γ		Rat (PC12, *in vitro*) (294) Bovine (chromaffin cells, *in vitro*) (295)		NFκB and STAT3 (295)
IL-4	Bovine (cortex) (296)			
IL-6	Human (cortex, medulla and chromaffin cells) (297, 298, 299) Rat (medulla and PC12 cells) (300, 286, 301, 302) Bovine (ZG, ZF, ZR, medulla, and chromaffin cells) (266, 271)	Human (adrenal, *in vitro*) (303, 299) Rat (PC12, *in vitro*) (304, 305)	IL-6R; Human (normal and macrophage-depleted adrenal) (303, 299), Rat (medulla) (300) Gp130; Human (adrenal) (303), Rat (medulla) (300)	c-Fos (305) STAT3(304)
IL-8	Human (cortex, H295R cells) (306) Bovine (chromaffin cells) (271)			
IL-10	Rat (PC12) (307)			
IL-11	Rat (PC12_bPAC1hop cells) (308)			
IL-15	Rat (medulla) (293)			
TNF-α	Human (ZR, medulla, chromaffin cells, and pheochromocytoma) (267, 297, 309) Rat (PC12) (286) Bovine (adrenal capsule, cortex, and medulla) (266)	Rat (PC12, *in vitro*) (310, 311, 312) Mouse (chromaffin cells, *in vitro*) (313) Bovine (chromaffin cells, *in vitro*) (271, 289, 314, 315, 291, 313, 316)	TNFR1; Bovine (chromaffin cells) (314) TNFR2; Bovine (chromaffin cells) (289, 314, 315)	ERK 1/2, p38, AP-1, NFκB (289) NFκB (RelA, NFκB1, NFκB2) (315) NFκB (various Rel class members) (271)
CCL2	Rat (medulla, PC12_bPAC1hop cells) (293, 308) Bovine (chromaffin cells) (271)			
CCL5	Bovine (chromaffin cells) (271)			
CCL7 CXCL14 TNFS8 NAMPT	Rat (PC12_bPAC1hop cells) (308) Bovine (chromaffin cells) (308) Bovine (chromaffin cells) (308) Rat (PC12_bPAC1hop cells) (308) Bovine (chromaffin cells) (308)			
CXCL2 CXCL8	Bovine (chromaffin cells) (271)			

*JAK, Janus kinase; STAT, Signal Transducer and Activator of Transcription; NO, Nitric Oxide; GC, Guanylyl Cyclase; NPY, Neuropeptide Y; ZG, Zona Glomerulosa; ZF, Zona Fasciculata; ZR, Zona Reticularis*.

### Cytokine signaling in chromaffin cells

Wherever they may originate, there is now strong evidence that cytokines profoundly influence the adrenal medulla by inducing changes in secretion, intracellular signaling, gene transcription, and translation (318). The cytokines most studied for their influence on adrenal chromaffin cell function include IFN-α, IL-1β, IL-6, and TNF-α. These cytokines have likely received particular attention because they are prominent mediators of the systemic acute phase inflammatory response.

IFN-α is a type I interferon and signals via the IFN-α receptor (IFNAR) complex, which includes IFNAR1 and IFNAR2 subunits. Transcript expression of IFNAR2 has been reported to increase in response to TNF-α treatment of bovine adrenal chromaffin cells (271). In many cells, binding of ligand to IFNAR induces activation of janus kinase (JAK)/signal transducer and activator of transcription (STAT) signaling, with the phosphorylation of STAT1 and STAT2, which dimerize to form two different transcriptional activator complexes (a STAT1 homodimer and STAT1-STAT2-IRF9 heterotrimer). IFN-α can also activate other members of the STAT family (319). Treatment of bovine chromaffin cells with IFN-α induces phosphorylation, increased expression, and nuclear translocation of STAT1 and STAT2 (268). Further, IFN-α induces an increase in STAT3 phosphorylation but only increases nuclear STAT3 in a small proportion of cells (268). IFN-α also induces ERK1/2 signaling downstream of PKC activation in chromaffin cells (268, 320). Similar to IL-1, IFN-α inhibits ACh-stimulated CA secretion from chromaffin cells (269). IFN-α also suppresses NE uptake by cultured bovine chromaffin cells (270). IFN-α induces PKC- and ERK1/2-dependent phosphorylation of TH at the serine (Ser)-31 site (no change in phosphorylation at Ser-19 or -40), a post-translational modification that is linked to increased TH protein stability and activity (268, 321, 322). ERK1/2 activation has also been reported to contribute to histamine and Ang II-induced increases in TH Ser-31 phosphorylation in bovine adrenal chromaffin cells (323, 324). Similar mechanisms of post-translational regulation of TH by ERK1/2 in adrenal chromaffin cells may be utilized by other ERK1/2-activating cytokines.

IL-1β increases protein levels of the CA biosynthetic enzyme TH and, like IFN-α, induces phosphorylation of TH, in this case at the Ser-40 site which decreases inhibitory feedback of CAs on TH activity (280, 325). Induction of TH phosphorylation by either IL-1β or IFN-α is transient (lasting <30 min) (268, 280). Long-term (24 h) incubation with IL-1β does increase total TH protein, while incubation with IFN-α has not yet been demonstrated to change TH protein level (268, 280). IL-1β-induced phosphorylation of TH at other Ser sites and the involvement of ERK1/2 signaling in IL-1β-induced TH regulation have not been investigated. IL-1 receptors IL-1R1 and IL-1R2 are both expressed by rat adrenal medullary cells (275, 293, 292). IL-1R1 is responsible for transmembrane signaling and IL-1R2 is a decoy receptor that acts as an endogenous inhibitor, like IL-1RA, to IL-1 signaling (326). IL-1 exists in two forms, IL-1α and IL-1β. Although they are structurally very different, both IL-1α and IL-1β bind to the IL-1Rs and the neurochemical effects of both forms are similar (248). The similarity in effects of IL-1α and IL-1β is observed in adrenal chromaffin cells as well (280–282). Stimulation of chromaffin cells with IL-1 can induce PKA, ERK1/2, nitric oxide (NO)/PKC, and NO/guanylyl cyclase intracellular signaling mechanisms (280–282). Some IL-1-induced effects in chromaffin cells rely on intermediate autocrine signaling by factors such as NPY and CRH. IL-1 induction of NPY is responsible for downstream activation of PKA/NO, as well as ERK1/2, PKC and guanylyl cyclase pathways (280). IL-1-induced CRH expression can trigger a signaling loop, where CRH stimulates chromaffin cells to produce more IL-1β (272, 283). Exposure to IL-1 can also cause increased expression of IL-1R1 in PC12 cells (292). An autocrine signaling loop utilizing IL-1 is supported *in vivo*. Intravenous injection of IL-1β has been reported to increase IL-1β and IL-1R1 mRNA levels in the medulla of rats (275).

IL-1 alone has been reported to stimulate CA release from cultures of primary adrenal chromaffin cells and from pheochromocytomas (272, 280, 282, 284, 290). A significant portion of IL-1 induction of CA secretion relies on intermediate autocrine signaling by NPY (280, 287). In contrast to basal application of IL-1, when combined with ACh, IL-1 has an inhibitory effect on chromaffin cell CA release (288). IL-1 may function in the homeostatic control of CAs, where in the absence of stimulation by other sources, IL-1 enhances CA secretion, but when other activators are present IL-1 dampens their effects on CA secretion. An auto-regulatory mechanism utilizing IL-1 is supported *in vivo*, as administration of cholinergic agonists increases IL-1 mRNA and decreases IL-1 protein stores in rat adrenals, suggesting enhanced IL-1 secretion in response to cholinergic stimulation (274, 276). Interestingly, it has been reported that medullary expression of IL-1R2 is increased by immobilization stress (293). If IL-1 is involved in homeostatic control of CAs, increased expression of the decoy receptor IL-1R2 may be a stress-specific response, whereby IL-1R2 prevents IL-1 from dampening CA release in response to a psychological stressor. Differential regulation of CA biosynthetic enzymes has been reported in response to long-term exposure to cold or immobilization stress (220, 327, 328).

IL-6 binds to a receptor complex consisting of IL-6 receptor (IL-6R) and glycoprotein (gp) 130 components. IL-6 binds to IL-6R, which can exist in either soluble or membrane-bound forms. The receptor-ligand complex then couples to the gp130 signal transducing component, promoting dimerization of gp130, facilitating downstream signaling (329). JAK/STAT3 and MAPK/ERK signaling are common IL-6 activated pathways and are induced in neurons exposed to IL-6 (330). IL-6-induced STAT3 signaling has been reported in PC12 cells, and both STAT3 and ERK1/2 signaling mechanisms are supported by preliminary investigations using bovine chromaffin cells (304, 318, 331). The signal transducing component of the IL-6R complex is shared with other IL-6 family cytokines, which, like IL-6, bind to ligand-specific receptors which then complex with a gp130. Therefore, it is unsurprising that adrenal chromaffin cells are also responsive to other IL-6 family cytokines (300). In immune cells, IL-6 activates AP-1 via a Ras-dependent MAPK signaling mechanism (332). Activation of this transcription factor may also occur in chromaffin cells, as IL-6 has been reported to induce increases in c-fos transcript in PC12 cells (305). In PC12 cells, high concentrations of IL-6 were found to have an inhibitory effect the basal CA-producing function, decreasing DA and NE release as well as TH protein (304).

TNF-α binds to the plasma membrane situated receptors TNF receptor (TNFR) 1 and TNFR2. Binding of ligand to these receptors can initiate signaling cascades that utilize numerous protein kinases and lead to the activation of two major transcription factors, AP-1 and NFκB (289, 333). Bovine chromaffin cells have been found to express TNFR1 and TNFR2; however, TNFR1 appears to be the more consistently expressed of the two (289, 314, 315). A detailed investigation by Ait-Ali et al. (289) found that TNF-α signaling in bovine chromaffin cells relies on ERK1/2 and p38 signal transduction mechanisms. Further, this study determined that activation of the transcription factor AP-1 occurs downstream of ERK1/2 activation, also finding that TNF-α induces NFκB transcription factor activity in an ERK1/2-independent manner. In chromaffin cells, TNF-α-induced NFκB signaling appears to involve primarily the p65 subunit, and possibly p52 and p50 subunits. Thus, transcriptional regulation likely occurs primarily via the p65 homodimer (315). The inhibitor of NFκB, PDTC, blocks the enhancement of the transcript levels of some genes by TNF-α (315). Later experiments demonstrated that a variety of Rel family transcription factors are likely involved in the transcriptional regulatory effects of TNF-α (271). TNF-α has been reported to induce and to modulate neuropeptide transcript and protein in bovine adrenal chromaffin cells (289, 291, 313). The effects of TNF-α on CA synthesis have not been thoroughly investigated. A study of cytokine modulation of hypoxic response found that TNF-α can inhibit hypoxic induction of TH transcript (310). An oligonucleotide microarray analysis of TNF-α-induced changes in bovine chromaffin cell transcriptome identified upregulation of PNMT transcript after long (48 h), but not short (6 h) exposures to the cytokine (315).

**Figure 4:**
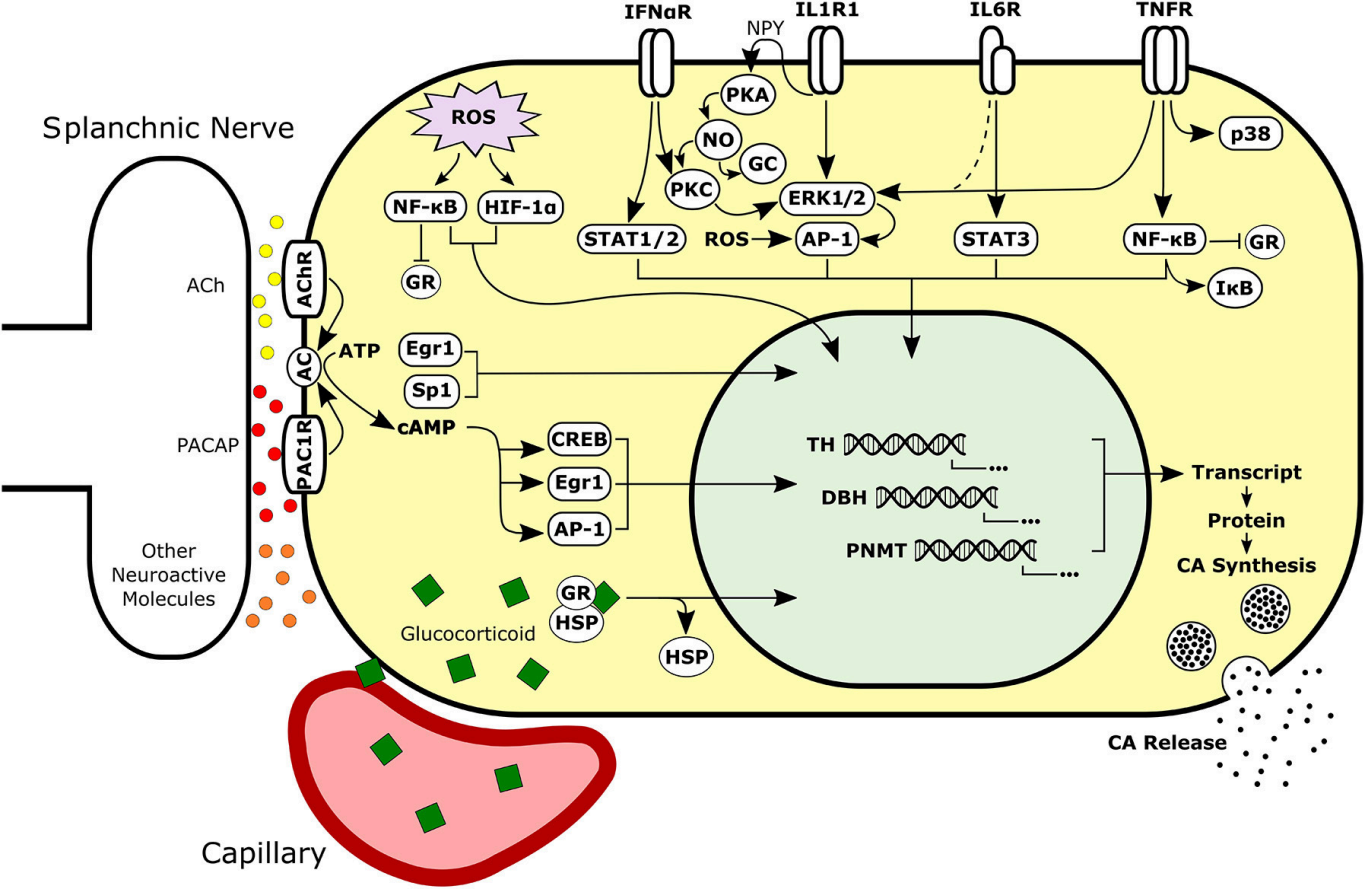
Simplified schematic of neural, hormonal, redox, and immune signaling pathways activated in adrenal chromaffin cells. Intracellular signals may be integrated to regulate synthesis and secretion of CAs by chromaffin cells during normal or pathological conditions. Chromaffin cells are responsive to extracellular signaling molecules in the adrenal such as glucocorticoid, cytokines, and neurotransmitters. Sympathetic neurons release neuroactive molecules including both small molecule neurotransmitters (e.g., ACh and ATP) and peptide neurotransmitters (e.g., PACAP and substance P). Dashed line represents signaling mechanism supported by unpublished findings. Ach, Acetylcholine; PACAP, Pituitary Adenylate Cyclase-Activating Peptide; AC, Adenylyl Cyclase; CA, Catecholamine; GR, Glucocorticoid Receptor; HSP, Heat Shock Protein; NPY, Neuropeptide Y; PKA, Protein Kinase A; NO, Nitric Oxide; PKC, Protein Kinase C; GC, Guanylyl Cyclase.

In addition to direct signaling mechanisms of cytokines (see Figure 4), evidence is now emerging that cytokines can induce long-term changes in chromaffin cells through the activation of autocrine signaling loops. It is a well-established phenomenon that in immune cells, cytokines favor their own synthesis and that of other cytokines, resulting in the formation of autocrine signaling cascades (334). Two long established examples are IL-1 and TNF-α, which can stimulate their own production, along with numerous other cytokines and inflammatory mediators (335–338). These autocrine signaling loops can be self-regulating by stimulating the production of anti-inflammatory molecules such as IL-10 (339, 340). By inducing the production of autocrine signaling molecules, cytokines may initiate long-term signaling programs in the adrenal medulla (266, 291, 313). For example, in primary cultures of bovine adrenal chromaffin cells, treatment with IL-1α has been reported to induce synthesis of the cytokines IL-6 and TNF-α, as well as the neuropeptides VIP, NPY, and Met-Enkephalin (266, 291). Intermediate autocrine signaling by NPY is critical for CA regulatory effects of IL-1 in chromaffin cells (280, 287).

Whether the responses of medullary cells to cytokines primarily functions for protective action against inflammatory stimuli or if cytokines are a normal part of the diverse informational molecules that constantly regulate chromaffin cell homeostatic function, local changes in cytokine signaling within the medulla have the potential to exacerbate dysfunctional CA synthesis. The regulation of adrenal function by cytokines and the importance of immune mechanisms in contributing to the progression of hypertension and CVD are summarized above. The bi-directional relationship of the immune and neuro-endocrine systems conceivably provides fitness advantages to organisms and may be a physiologically important part of maintaining health, dysfunction of which may result in pathology. The “neuro-immune circuit” has helped to explain perplexing phenomena such as the co-morbidity of neuropsychiatric symptoms and inflammatory disease (341). Similarly, integration of immune and adrenal functions provides an explanation for the etiology of inflammation-related hypertension and may help to elucidate mechanisms of essential hypertension.

### Cytokine modulation of glucocorticoid signaling in chromaffin cells

GCs and transmitters released at splanchnic-adrenal medullary synapses are important informational molecules which control Epi biosynthesis during normal and stress conditions [see (115) and references therein]. Chromaffin cells must coordinate intracellular signaling pathways induced by these and other informational molecules in order to produce appropriate responses under diverse physiological conditions. Cytokines produced either systemically or locally may be significant modulators of adrenal CA biosynthesis by altering chromaffin cell response to GCs and neurotransmitters. How, and to what extent, chromaffin cells simultaneously process information from immune and stress circuits is not well-understood.

A number of cytokines, including IFN-α, IL-1, IL-2, and TNF-α, have been reported to have inhibitory effects either on GC-induced GR nuclear translocation, GR-GRE binding, or GR-mediated gene transcription in diverse cell types (342, 343). In mouse hippocampal HT22 cells, inhibition of GR transcriptional activity by IFN-α is dependent on JAK/STAT signaling pathway. STAT5 (not STAT1 or STAT2) appears to be the major mediator of IFN-α inhibitory effects on GR function in HT22 cells. Co-immunoprecipitation revealed that phosphorylated STAT5 binds to GR within the nucleus, and IFN-α-induced repression of GR function does not rely on inhibition of GR protein expression or nuclear translocation (344). In bovine chromaffin cells, IFN-α does not induce phosphorylation of STAT5; instead, IFN-α primarily activates STAT1 and STAT2 (268). A trimeric complex consisting of STAT1, STAT2, and interferon regulatory factor (IRF) 9, induced by IFN-α/β receptor activation, is directly regulated by the nuclear coactivator of GR, glucocorticoid receptor-interacting protein (GRIP) 1 in murine macrophages (345). Occupancy of target promoters by the STAT1-STAT2-IRF9 complex, pre-initiation complex assembly, and type I IFN stimulated gene expression were reported to be repressed by dexamethasone (Dex)-induced depletion of GRIP. Inhibition of IFN-induced gene expression by Dex appears to be caused by sequestration of GRIP1 from its functional activity as a coactivator for the STAT1-STAT2-IRF9 complex by GR. Conceivably, as long as GRIP1 protein levels are sufficiently low in adrenal chromaffin cells, this competitive mechanism could allow for the converse effect, whereby induction of the STAT1-STAT2-IRF9 complex by IFN-α leads to an inhibition of GR transcriptional effects through the repression of GRIP1 coactivation of GR.

In mouse fibroblast cells, inhibition of GR-GRE binding and GR-mediated promoter activation by IL-1 has been reported to depend on p38 signaling (346). The mechanism of p38 regulation of GR appears to involve direct phosphorylation of GR at the Ser-211 site (human) which modulates recruitment of GR coactivators to GRE-containing promoters (346, 347). So far, p38 signaling has not been demonstrated as a significant contributor in IL-1 regulation of chromaffin cell function (288). Activation of p38 signaling pathway could possibly be a mechanism for inhibition of GR function by other cytokines, such as TNF-α, which have been reported to induce p38 signaling in chromaffin cells (289).

IL-2 inhibition of GR is dependent on signal transduction by p38 (reported in murine HT-2 T-helper cell line) and by JAK3 (reported in human PBMCs), both contributing to inhibition of GR nuclear translocation (348, 349). In a murine T-lymphocyte cell line (CTLL-2), IL-2 represses GR transactivation through direct protein-protein interactions of IL-2-induced STAT5 with GR following translocation to the nucleus (350).

In human keratinocyte-derived HaCaT cells, TNF-α inhibits GC-induced transcriptional activation and GR nuclear translocation through a MEK-1/ERK-dependent mechanism (351). In human epithelial-derived HeLa cells, the inhibitory effect of MAPK signaling on GR function is demonstrated by inhibition of transcriptional activation of GR-responsive luciferase constructs in the presence of constitutively activated p38 or JNK (352). In human colon carcinoma-derived HCT116 cells, TNF-α can suppress GR transactivation by induction of FLICE-associated huge protein, which competes with GR to bind the nuclear coactivator GRIP1 (353). Additionally, mutual repression of GR and NFκB function may result from transrepression (also known as tethering), caused by direct protein-protein interactions within the nucleus, or from competition for the cofactors CBP and steroid receptor coactivator-1 (354–356).

IL-6 enhances GR function through a STAT3-dependent mechanism in rat hepatoma H4IIE cells. Like STAT5, STAT3 forms complexes with GR in the nucleus; however, unlike STAT5, formation of a STAT3-GR complex appears to enhance promoter activation, both of IL-6 responsive elements and of GREs (357).

IL-10 is another cytokine reported to have synergistic rather than inhibitory effects on GR function. In a study using human leukocytes (U937 cells and whole blood cell cultures), treatment with the anti-inflammatory cytokine IL-10 had opposite effects to TNF-α. Inhibition of IL-6 secretion in whole-blood cell cultures and promotion of IL-1R antagonist secretion by human monocyte-derived U937 cells are two effects of Dex treatment. TNF-α supresses, whereas IL-10 intensifies these Dex-induced activities (358). The mechanism of IL-10 and GR signaling crosstalk is not yet understood; however, the effect may be achieved through an increase in GR expression (358). Other possible mechanisms include IL-10-induced activation of STAT3, or IL-10-induced inhibition of NFκB, decreasing the transrepression effect (355, 359, 360).

The mechanisms of cytokine-induced GR regulation involve intermediate signaling molecules such as MAPKs and transcription factors including STAT1, STAT2, STAT3, STAT5, NFκB, and AP-1. In chromaffin cells, the most commonly reported signaling pathway by cytokines is that of the MAPK transduction molecules, particularly ERK1/2 (318). The MAPKs JNK and ERK can inhibit GR-mediated transcriptional activation by direct and indirect phosphorylation of GR at Ser-246 in rats (Ser-226 in humans), which may prevent signaling by promoting nuclear export of the transcription factor (361, 362). ERK1/2 signaling can also lead to AP-1 transcription factor activity. In chromaffin cells, both IL-6 and TNF-α have been reported to induce activation of AP-1 subunits (289, 305). Treatment of bovine chromaffin cells with TNF-α increases transcriptional activation of AP-1 responsive promoter elements and increases binding of fos and jun proteins to TPA-responsive element (TRE) and CRE sequences through an ERK1/2-dependent mechanism (289). In humans, GC resistance is positively correlated with expression of c-fos in peripheral blood mononuclear cells *in vivo* (363). The interaction between GR and AP-1 is mutually repressive, involving tethering at the GR-DNA binding domain; thus, inhibition of GR function may occur after GR translocation to the nucleus (364–367). Activation of AP-1 signaling may be a component of chromaffin cell regulation by other ERK1/2-inducing cytokines, such as IFN-α and IL-1, and leading to insensitivity of chromaffin cells to GCs.

### Cytokine modulation of neurotransmitter and cAMP signaling in chromaffin cells

Signal integration of neurotransmitters such as ACh and PACAP with some cytokines has been demonstrated in chromaffin cells. IL-1 inhibits ACh-induced CA release via reducing Ca^2+^ influx in bovine chromaffin cells; this is triggered by ERK1/2 signaling pathways (288). Similarly, IFN-α has been reported to inhibit ACh-induced CA secretion and Ca^2+^ influx in bovine chromaffin cells (269). Chromaffin cell response to the neuropeptide PACAP is also modified by cytokine exposure. Combined treatment with PACAP and TNF-α synergistically upregulates VIP and galanin expression in bovine chromaffin cells (313).

The signaling pathways of cAMP and cytokines may be integrated through interactions at MAPK, JAK-STAT, and NFκB. A number of studies report effects of cAMP/PKA signaling on the function of these other signaling molecules. In a human T-lymphocyte cell line (Jurkat cells) cAMP increases ERK1/2 activity by inhibiting function of haematopoietic protein tyrosine phosphatase, a negative regulator of ERK1/2 and p38 (368). In experiments using mouse (70Z/3) and human (Jurkat) lymphocyte cell lines, PKA was reported to contribute to the activation of NFκB by phosphorylating p65 following dissociation from IκB (369). In human PBMCs, cAMP has an inhibitory effect on IL-6-induced STAT1 and STAT3 binding to DNA and prevents IL-6 induction of an IL-6-responsive gene harboring a STAT1 and STAT3 binding sequence (370). Compared to cAMP regulatory effects on cytokine signaling pathways, the effects of cytokines on cAMP signaling are less well-understood. This may in part be because cAMP activation occurs early in intracellular signaling cascades and because the cellular output of cAMP activation is highly tissue-dependent (371, 372). In chromaffin cells, transcription factors involved in the response to cAMP/PKA signaling include Egr1, AP-1, and CREB (202, 203, 209). Signaling via cAMP in chromaffin cells may also lead to activation of the MAPKs p38 and ERK1/2 (200, 201). In bovine chromaffin cells, the cytokines IL-1 and TNF-α have been reported to enhance induction of VIP and substance P expression by forskolin (Forsk), an activator of adenylyl cyclase (291). The mechanisms of cytokine modulation of cAMP signaling in chromaffin cells have not been defined.

### Free radicals, cytokines, and hypertension

Oxidative stress and reactive oxygen species (ROS) are active contributors to the pathogenesis of hypertension and CVD. For example, ROS-mediated signaling and activation of CA biosynthesis have been associated with elevated BP in patients who suffer from obstructive sleep apnoea and as such experience intermittent hypoxia (IH) (373, 374). Adrenal derived cells exposed to IH or sustained hypoxia show ROS-mediated elevation in CA secretion, upregulation in TH, DBH and PNMT, driven by HIF-1α, Egr1, and Sp1 (375–384). Furthermore, numerous studies have highlighted the interrelationship between the immune response and oxidative stress in organs such as the heart, brain, or the kidney, ultimately leading to vessel and organ damage, and hypertension (64, 385, 386). Oxidative stress can be a major contributor of immune activation in hypertension via an autoimmune-like reaction driven by isoketals (products of ROS-mediated fatty acid oxidation) that serve as auto- antigens (106). Like HIF-1α, the transcription factor NFκβ (a transcription factor with complicated roles in inflammatory signaling pathways) is oxygen-sensitive, and release of mitochondrial H_2_O_2_ has been demonstrated to activate it (387, 388). Activation of NFκβ, Nrf2 or other proinflammatory transcription factors via ROS can modulate the biosynthesis of proinflammatory cytokines, and other molecules like VEGF, which can affect vascular damage associated with hypertension. Moreover, studies report a correlation of BP with circulating cytokines, and oxidative stress parameters; proinflammatory cytokines can lead to more ROS generation perpetuating the effects on the hypertensive state (389). For example, treatment with AngII inhibitors lowered pro-inflammatory cytokines and reduced parameters of oxidative stress in hypertensives, while dietary antioxidant intervention leads to lowered inflammatory markers such as CRP and IL-6, and improvement in BP (69, 390–392).

## Concluding remarks

Numerous cytokines regulate expression of enzymes responsible for biogenesis of CAs, the major secretory product of chromaffin cells and important regulators of BP homeostasis. Constitutively expressed cytokines may have an important function in homeostatic control of CA biosynthesis and may modify CA biosynthesis during inflammation. Further, adrenal regulation by cytokines could be an important innate mechanism for preventing the progression of hypertension, by dampening CA biosynthesis with the development of inflammation. Additionally, the inhibition of GC-induced adrenal medullary activation by cytokines may be part of an autoregulatory loop to prevent medullary over-stimulation particularly when inflammation induces a compensatory increase in GC secretion (an important endogenous anti-inflammatory molecule) (393). Increased concentration of GCs in the adrenal medulla, in the absence of such an inhibitory mechanism, would result in increased CA release (394). Thus, immune changes that coincide with hypertension could signal an adaptive inhibition of CA biosynthesis, preventing adrenal medullary over-activation through cytokine-mediated antagonism of GC-induced chromaffin cell activation. Both effects may be protective mechanisms against the development of hypertension; disturbance of such mechanisms, either by changes in local adrenal cytokine concentrations or by disruption of chromaffin cell sensitivity to cytokines, could be contributing factors to the progression of hypertension. Future investigations to determine changes in local cytokine concentrations in the adrenal medulla during prehypertension and overt hypertension will provide better insight into the relevance of cytokine-chromaffin cell signaling in this disease. Moreover, in addition to their effects in the adrenal, many cytokines also modulate CA levels in the hypothalamus and influence function of the HPA axis, and conceivably the neuro-endocrine circuit (248, 249).

The microenvironment of the adrenal gland is a viable locale for cross talk between endocrine pathways and immune response networks (395). Intermediary signaling molecules like ROS could be involved in integration of the signaling networks. Overall, the molecular mechanisms involved in integrating hormonal, neural, immune, and possibly redox inputs to the adrenal medulla remain to be elucidated. The patterns of inter-adrenal cytokine regulatory effects on CA enzyme expression could provide insight into potential converging points of interaction between these pathways (Figure 4). These networks involve direct or indirect, bidirectional interactions at the cellular and/or molecular levels of CAs, GCs, cytokines, and ROS. Understanding these unique interactions will help to improve our current understanding of adrenal functioning and HPA regulation in hypertension.

## Author contributions

CB, SK, AK, and TT contributed conception and structure and focus of the review manuscript; CB and SK compiled published reports and manuscripts relevant to the review and provided summaries and wrote the first draft of the manuscript; AK and TT provided critical reviews and edits of manuscript versions. All authors contributed to manuscript revision, read and approved the submitted version.

### Conflict of interest statement

The authors declare that the research was conducted in the absence of any commercial or financial relationships that could be construed as a potential conflict of interest.

## Abbreviations

AADC: Aromatic amino acid decarboxylase
ACh: Acetylcholine
ACTH: Adrenocorticotropic hormone
Ang: Angiotensin
AP-1: Activator protein 1
AP-2: Activator protein 2
BP: Blood Pressure
CA: Catecholamine
CBP: CREB-binding protein
CNS: Central nervous system
CRE: cAMP response element
CRH: Corticotropin-releasing hormone
CVD: Cardiovascular disease
DA: Dopamine
DBH: Dopamine β-hydroxylase
DBP: Diastolic blood pressure
DOCA: Deoxycorticosterone acetate
Egr1: Early growth response 1
Epac: Exchange protein directly activated by cAMP
Epi: Epinephrine
ERK: Extracellular signal regulated kinases
GC: Glucocorticoid
GRE: Glucocorticoid response element
GRIP: Glucocorticoid receptor-interacting protein
HPA: Hypothalamic-pituitary-adrenal
IFNAR: IFN-α receptor
IL-1R: IL-1 receptor
IL-6R: IL-6 receptor
IRF: Interferon regulatory factor
JAK: Janus kinase
LDCV: Large dense core vesicle
L-DOPA: L-3,4-dihydroxyphenylalanine
MAPK: Mitogen-activated protein kinase
NE: Norepinephrine
NO: Nitric oxide
PACAP: Pituitary adenylate cyclase-activating peptide
PKA: Protein kinase A
PKC: Protein kinase C
PLC: Phospholipase C
PNMT: Phenylethanolamine N-methyltransferase
RAAS: Renin-angiotensin-aldosterone system
SA: Sympathetic-adrenal
SBP: Systolic blood pressure
Ser: Serine
SHR: Spontaneously hypertensive rat
SNS: Sympathetic nervous system
Sp1: Specificity protein 1
SSV: Small synaptic vesicle
STAT: Signal transducer and activator of transcription
TH: Tyrosine hydroxylase
TNFR: TNF receptor
TRE: TPA-responsive element.
